# Performance of Dairy Cows Offered Grass Silage Produced within Either a Three- or Four-Harvest System When Supplemented with Concentrates on a Feed-to-Yield Basis

**DOI:** 10.3390/ani13020228

**Published:** 2023-01-07

**Authors:** Aimee-Louise Craig, Alan W. Gordon, Conrad P. Ferris

**Affiliations:** 1 Agri-Food and Bioscience Institute Hillsborough, Large Park, Hillsborough BT26 6DR, UK; 2 Agri-Food and Bioscience Institute Newforge, Newforge Lane, Belfast BT9 5PX, UK

**Keywords:** dairy cattle, multi-harvest silage, silage feed value, silage systems, precision feeding

## Abstract

**Simple Summary:**

Harvesting grass for silage at an earlier growth stage can improve silage quality and subsequent dairy cow performance. This study investigated the impact of more frequent harvesting of grass for silage production on dairy cow performance in a system offering concentrates on a feed-to-yield basis. Grass silage was harvested either three- (3H) or four- (4H) times during the summer and then offered to lactating cows for 25 weeks post-calving. Cows were offered their respective silage treatment as part of a mixed ration containing 8 kg of concentrate per cow per day. Cows were also offered additional concentrate on a feed-to-yield basis according to individual cow milk yields. The silage produced under 4H had higher energy and protein content than silage harvested within the traditional 3H system. The increase in silage quality with 4H resulted in an increase in silage intake, milk yield and milk protein content. This study has demonstrated that increasing silage harvesting frequency can improve silage quality and subsequent animal performance.

**Abstract:**

More frequent harvesting of grass swards provides an opportunity to improve the nutritive value of grass silage. This study investigated the effect of offering silages produced within either a three- (3H) or four-harvest (4H) system on dairy cow performance when concentrate supplements were offered according to the individual cow’s milk yield (feed-to-yield). Cows (n = 80) were allocated to either 3H or 4H at calving and remained on experiment for 25 weeks. Within both treatments, cows were offered silage from each harvest consecutively in proportion to the dry matter (DM) yield for each harvest. Silage was offered as a mixed ration with concentrate added at a rate of 8 kg/cow/day. Additional concentrates were offered on a feed-to-yield basis. Herbage yields were reduced in the 4H system, but 4H silage contained higher metabolisable energy and crude protein content compared to 3H. Cows offered the 4H silage had greater silage DM intake, milk yield and milk protein content, while milk fat content was greater in cows offered 3H silages. In conclusion, increasing harvesting frequency from three to four harvests per year can improve silage feed value, silage intakes and milk yields when concentrates are offered on a feed-to-yield basis.

## 1. Introduction

Grass silage is the predominant forage offered to housed dairy cows during the ‘winter’ months in many northern European countries, including western parts of the United Kingdom (UK) and Northern Ireland (NI). As herd sizes increase and dairy farming becomes more intensive, cows are being housed for longer periods, or housed full-time, which increases reliance on grass silage in these regions. Harvest date is the most important factor affecting silage digestibility [1] due to the increased degree of lignification of fibre with increasing plant maturity [2]. For example, silage digestible organic matter in the dry matter (D-value) declines by an average of 3.3% with each one week delay in harvest date [1]. Furthermore, for each 10 g/kg increase in silage dry mater (DM) digestibility, dry matter intake (DMI) and milk yield increase by 0.22 kg/day and 0.33 kg/day, respectively [1].

While the impact of silage feed value on cow performance has been examined in many studies, these have often involved silages made from primary growth herbage. However, on commercial farms, silages made from primary, secondary and indeed third regrowth herbage is often offered. While regrowth herbage is leafier than primary growth herbage [3], it frequently contains a higher proportion of indigestible NDF than the latter [4]. This highlights the need to examine the impact of the whole season harvesting system on cow performance, including the impact of harvesting earlier and more frequently. The impact of harvesting frequency was examined by Ferris et al. [5] two decades ago, and this study demonstrated that concentrate inputs were reduced by an average of 55% without loss in milk production when cows were offered silages produced within a four- compared to a two-harvest system. However, since this work was undertaken, the majority (65%) of farmers in NI have moved to a three-harvest system [6], while milk yields have also increased considerably due to improved cow genetics and increased concentrate feed levels. With regards to the latter, a large proportion of NI dairy farmers now offer concentrates on a ‘feed-to-yield’ (FTY) basis, rather than on a ‘flat-rate’ basis, as was adopted by Ferris et al. [5]; however, milk yield responses of cows offered concentrates on an FTY basis are known to differ from responses when a traditional ‘flat-rate’ approach is adopted [7,8].

Consequently, the current study was designed to evaluate the responses of higher yielding dairy cows when offered silages produced within two contrasting systems, and when supplemented with concentrates using an FTY approach. The authors recognise that offering concentrates using an FTY approach means that concentrate levels are not constant with each silage type, but rather are determined by actual cow performance within each silage, relative to average energy intakes from the basal diet. Furthermore, each silage was supplemented with a concentrate specifically formulated to complement that silage. These approaches were adopted to ensure that the research findings were relevant to progressive dairy farms where these strategies are already practiced.

## 2. Materials and Methods

This study was conducted at the Agri-Food and Biosciences Institute (AFBI), Hillsborough, NI (54°27′ N; 06°04′ W).

### 2.1. Experimental Animals

This study involved 80 Holstein dairy cows, 24 primiparous and 56 multiparous (mean lactation number 2.8 (SD 1.77)), with a mean calving date of 1 November 2018 (range, 5 October to 12 December 2018). Cows had a mean predicted transmitting ability (PTA2018) for milk yield, fat yield and protein yield of 251 (SD 157.9) kg, 14.9 (SD 5.4) kg and 14.0 (SD 4.7) kg, respectively. During the three-week period pre-partum, cows were given *ad-libitum* access to grass silage supplemented with a pre-calving mineral/vitamin mix and with calcined magnesite to achieve target intakes of 100 and 50 g per cow/day.

Cows were moved to a free-stall house within 24 h post-calving. Two treatments, comprising silage produced within either a three-harvest system (3H) or a four-harvest system (4H), were examined. Cows were allocated to each of the two silage treatments at calving (28 multiparous and 12 primiparous cows per treatment). Treatment groups where balanced for calving date, lactation number, PTA for milk yield, milk fat yield and milk protein yield, body weight (BW) and body condition score (BCS) at calving, and in the case of multiparous cows, previous 305-day milk yield.

### 2.2. Sward Management

The silages offered were produced from perennial ryegrass (*Lolium perenne* L.) based swards, with target intervals between harvests of approximately 50 days with the 3H system and 35 days with the 4H system. Actual cutting dates for the 3H system were 29 May, 24 July, and 11 September (harvests one to three, respectively), while actual cutting dates for the 4H system were 17 May, 25 June, 8 August and 11 September 2018 (harvests one to four, respectively).

Swards used to produce the silages were located within six adjoining fields (total area of 16.8 ha). Each of the four largest fields were divided into two halves, with one half managed according to a three-harvest system while the other half was managed according to a four-harvest system. The two remaining smaller fields were allocated at random, one each to the three- and four-harvest system. Both systems where managed so that whole season applications of total available N (organic + inorganic N) were similar (300 and 310 kg N/ha with the 3H and 4H systems, respectively). Swards were harvested using a Class 3200 mower (Harsewinkel, Ostwestfalen-Lippe, Germany), tedded when possible to facilitate wilting (duration of wilting periods ranged from 18–26 h for all harvests except the final harvest with each system, when the duration of wilting was approximately 40 h), placed into rows using a Class 3100 grass rake (Harsewinkel, Ostwestfalen-Lippe, Germany), and harvested using a John Deere 7450 precision-chop forage harvester (Moline, IL, USA). Grass was treated at harvest with a bacterial inoculant (Super MV-50, Biotal, Worcestershire, UK; containing 200 × 10^12 cfu/kg^ of *Lactiplantibacillus plantarum*, *Pediococcus acidilactici*, *Lacticaseibacillus paracasei*) at approximately 20 mL per tonne of fresh herbage, before being ensiled in separate bunker silos (100–200 tonne capacity), covered in polythene sheeting, and the sheet weighed down with rubber mats. While the target DM for herbage at harvest was approximately 300 g/kg, this was not always achieved due to extreme weather conditions and harvesting delays. In particular, high temperatures and unplanned harvesting delays while the second harvest within the 4H system was wilting resulted in a high DM content. However, rainfall during harvest three of the 4H system led to a low DM content. Furthermore, the final harvest of both systems occurred in September when a 24 h wilt was unable to achieve the target DM content of 300 g/kg due to reduced sunlight hours and lower air and ground temperatures.

The fresh weight of herbage harvested at each of the seven harvests during the study was determined by weighing all trailer loads of grass on a commercial weighbridge. A herbage sample was taken from throughout each load of herbage after it had been emptied from the trailer and the sample dried at 85 °C for 24 h to determine oven DM content. The yield of herbage DM harvested at each harvest within each of 3H and 4H was subsequently determined.

### 2.3. Experimental Diets

Each cow was offered each of its treatment silages consecutively (i.e., harvest one, followed by harvest two, etc.) for a target number of days, with the change from one silage to another taking place on a single day each week. The target number of feeding days for each silage was in proportion to the herbage DM yield for each harvest. Target feeding days for harvests one to three within the 3H treatment were 70, 55, and 57 days, respectively, while target feeding days for harvests one to four within the 4H treatment were 52, 44, 41 and 45 days, respectively. However, silage shortages (due to higher than anticipated intakes) of the final silage offered within 3H and 4H treatments meant that the study was reduced in length to 175 days (25 weeks).

Diets were designed to meet the cows metabolizable energy (ME) and metabolizable protein requirements (MP), according to the equations within Feed-into-Milk, the dairy cow feeding system currently adopted within the United Kingdom [9]. Rations were formulated using NutriOpt software (Nutreco, Amersfoort, The Netherlands), which, in seeking to meet MP requirements, takes account of fermentable energy, total fermentable carbohydrates and protein balance. Rations contained a range of common ingredients as per practice on-farm. The grass silage component of the diet was mixed with a concentrate to form a partial mixed ration (basal ration). The concentrate fraction was included in the mix at a rate of 8.6 kg per cow/day, to achieve a target concentrate intake of 8.0 kg per cow/day (rations offered *ad libitum* at 107.5 % of the previous day’s intake). A separate concentrate was formulated for each silage type, with the ingredient list and chemical composition of each concentrate presented in Table 1. The rations were prepared daily using a mixer wagon (Vari-Cut 12, Redrock, Armagh, UK) and offered between 09.00 and 10.00 h, while uneaten food was removed the following day at approximately 08.00 h. The appropriate silage was placed in the wagon, and mixed for approximately five minutes, after which the appropriate concentrate was added to the wagon and the silage and concentrate mixed for a further five minutes. The ration was transferred from the mixer wagon to a series of individual feed boxes mounted on weigh scales (Controlling and Recording Feed Intake, Bio-Control, Rakkestad, Norway). Cows were given access to these boxes via an electronic identification system, and individual cow intakes were recorded daily.

Cows were offered additional concentrates (ingredient list and chemical composition in Table 1) on an FTY basis, with 2.0 kg/day of this concentrate offered via an in-parlour feeding system (fixed throughout the duration of the study; 1.0 kg at each milking) and the remainder offered via an out-of-parlour feeding system (OPF). Concentrates offered via the OPF increased during the first 21 days post-calving by 0.25 kg/day (from 0 to 5.25 kg/day) for multiparous cows and by 0.20 kg/day (from 0 to 4.2 kg/day) for primiparous cows. These concentrate feed levels remained unchanged until 28 days post-calving, after which these concentrates were offered on an FTY basis. Concentrate levels were reviewed weekly (on the same day that cows moved from one silage to another) and adjusted on the basis of milk yields during the previous two weeks. The first step in the process involved determining average daily silage and concentrate intakes from the basal ration (on a group basis, recorded using the feed intake system) over the previous 14 day period. Total ME intake from the basal ration was then determined based on the predicted ME concentration of the silage offered (based on weekly analysis), and the estimated ME concentration of the concentrate (based on formulated values, NutriOpt). This intake was assumed to support the cow’s maintenance energy requirement (based on equations in Feed-Into-Milk [9]), plus the production of a certain amount of milk (based on an assumed ME requirement of 5.2 MJ/kg milk). The milk produced by each cow not supported by the basal ration was determined as the difference between the actual milk yield over the previous two weeks, and the milk yield that the basal ration was calculated to support. Concentrates were offered at a rate of 0.45 kg, for each kg of milk not supported by the basal ration. The basal ration in the 3H treatment (excluding the build-up period) was calculated to provide sufficient ME to meet the cow’s maintenance energy requirements plus 23.8 (19.1), 24.1 (19.4), and 23.6 (18.8) kg milk/day for cows (heifers) offered silage from harvests one to three, respectively. Similarly, excluding the build-up period, the basal ration in the 4H treatment was calculated to provide sufficient energy to meet the cow’s maintenance energy requirements plus 26.2 (21.2), 26.1 (20.9), 25.9 (20.9) and 26.0 (20.8) kg milk/day for cows (heifers) offered silage from harvests one to four, respectively. If individual cow milk yields fell below the yield which the basal ration was able to support, concentrate levels offered through the OPF were held at 1.0 kg per cow/day for three weeks, and thereafter concentrate feeding via the OPF ceased.

### 2.4. Cow Measurements

Throughout the experimental period, cows were milked twice daily (between 06.00 and 08.00 h and between 15.00 and 17.00 h) using a 50-point rotary milking parlour (Boumatic, Madison, WI, USA). Individual cow milk yields were automatically recorded at each milking, and a daily milk yield for each 24 h period calculated. Each week a milk sample from each cow was taken during two consecutive milkings, analysed for fat, protein and lactose concentrations using an infrared milk analyser (Milkoscan CombifossTM7; Foss Electric, Hillerød, Denmark), and a weighted concentration of each constituent calculated for the 24 h sampling period.

Individual cow BW was recorded using an automated weigh bridge twice daily (immediately after each milking), and a mean weekly BW for each cow was determined. The BCS of individual cows was estimated on a five-point (including quarter points) scale by the same trained technician each fortnight, according to Edmonson et al. [10]. The daily EB (MJ of ME/d) for each cow was calculated using equations contained within Feed-Into- Milk [9], as the difference between the cow’s total ME requirements (maintenance, milk production, and activity) and total ME intake. Blood samples were collected from the tail of each cow prior to feeding at 4, 8, 12, 16 and 20 weeks of lactation, and centrifuged (3000 rpm for 15 min) to isolate either the serum (tubes with a clot activator) or the plasma (fluoride oxalate tubes). Serum beta-hydroxybutyrate (βHB), non-esterified fatty acids (NEFA) and urea concentrations, and plasma glucose concentrations were determined as described by Little et al. [11].

### 2.5. Feed Analysis

Each day a sample of the grass silage was taken from throughout the pile of mixed silage and dried at 85 °C for 18 h to determine oven DM content. At two time points in each week, a sample of grass silage was dried at 60 °C, and this was bulked for each fortnight, milled through a sieve with 0.85 mm aperture and subsequently analysed for neutral detergent fibre (NDF), acid detergent fibre (ADF) and ash. A fresh silage sample was analysed on a weekly basis for ME concentration using NIRS [12]. Furthermore, a fresh silage sample was also analysed on a weekly basis for gross energy, nitrogen (N), pH, ammonia-N, lactic acid and volatile components. A weekly sample of each concentrate offered was collected. A sub-sample was dried at 85 °C for 24 h to determine oven DM content, while a second sub-sample was dried at 60 °C for 48 h, bulked for each fortnight, milled through a 0.85 mm sieve, and subsequently analysed for N, NDF, ADF, ash and starch. All chemical analyses of the feedstuffs offered where undertaken as described by Purcell et al. [8].

### 2.6. Statistical Analysis

Weekly data for DMI, milk yield, milk composition, BW, and calculated EB, fortnightly data for BCS, and periodic blood metabolite data (4, 8, 12, 16 and 20 weeks), were analysed using REML repeated measures analysis with week post-calving included as the repeated measure. The model included the following terms as fixed effects: Lactation number + week + treatment + (week × treatment). Cow within week was also included as a random effect. The correlation between weeks was modelled using an autoregressive model of order 1. In addition to lactation number, PTA for milk yield, fat yield, protein yield, fat plus protein yield and milk composition were used as covariates in the analysis of the corresponding variables. All data were analysed using GenStat (19.1; VSN International Limited, Oxford, UK).

## 3. Results

Herbage DM yields at harvests one, two and three in the 3H system were 5.2, 4.1 and 4.1 (total, 13.4) t DM/ha, while the corresponding values at harvests one, two, three and four within the 4H system were 3.4, 3.6, 2.6 and 2.8 (total, 12.3) t DM/ha. The oven DM content of herbage at ensiling, and the chemical composition of the silages produced, are presented in Table 2. Dry matter of herbage ensiled, and of the resultant silages, varied between harvests within systems, reflecting the variability of weather conditions encountered during the season. Silage protein levels tended to increase from first through to the last harvest within each system. When silage composition within each harvest was ‘weighted’ using herbage DM yield within that harvest, mean CP, NDF and ME concentrations over all harvests were 143 g/kg DM, 519 g/kg DM and 10.7 MJ/kg DM respectively for silage within the 3H treatment, and 164 g/kg DM, 472 g/kg DM and 11.3 MJ/kg DM for silage within the 4H treatment.

**Figure 1:**
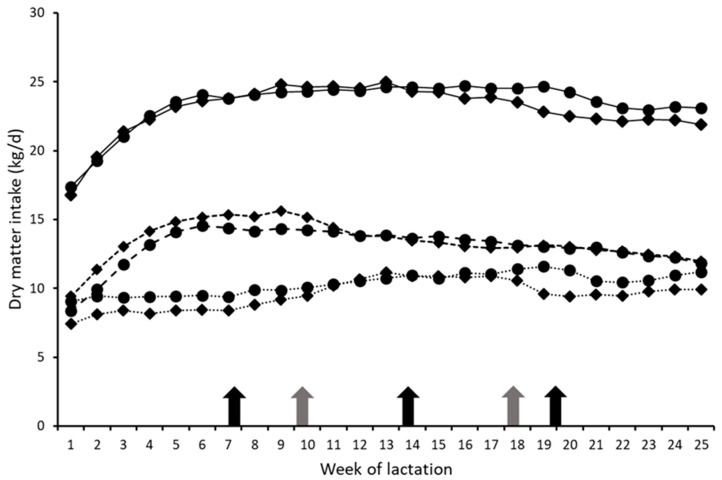
Mean weekly silage DMI (dotted lines), concentrate DMI (dashed lines) and total DMI (solid lines) of cows offered silage produced within either a three- (3H; ◆) or four-harvest (4H; •) system. Arrows indicate when cows changed to harvests 2,3 and 4 (4H; black arrow) and harvests 2 and 3 (3H; grey arrows).

Mean silage DMI were 8.5, 10.9 and 9.7 kg/d (Harvests 1–3, 3H treatment) and 9.3, 10.3, 11.2 and 10.8 kg/d (Harvests 1–4, 4H treatment), while the respective values for total DMI were 22.7, 24.4 and 22.5 kg/d and 21.8, 24.3, 24.5 and 23.4 kg/d. Average silage DMI across all harvests was greater for cows on the 4H treatment than for those on the 3H treatment (*p* < 0.001), while concentrate and total DMI were unaffected by treatment (Table 3). Intakes of all parameters varied over time (Figure 1, *p* < 0.001), and there was a significant interaction (*p* < 0.001) between treatment and week for silage DMI (Table 3).

**Figure 2:**
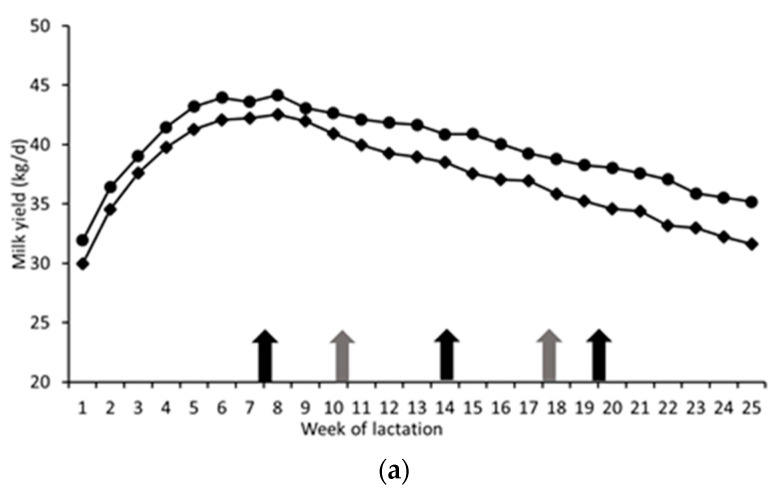

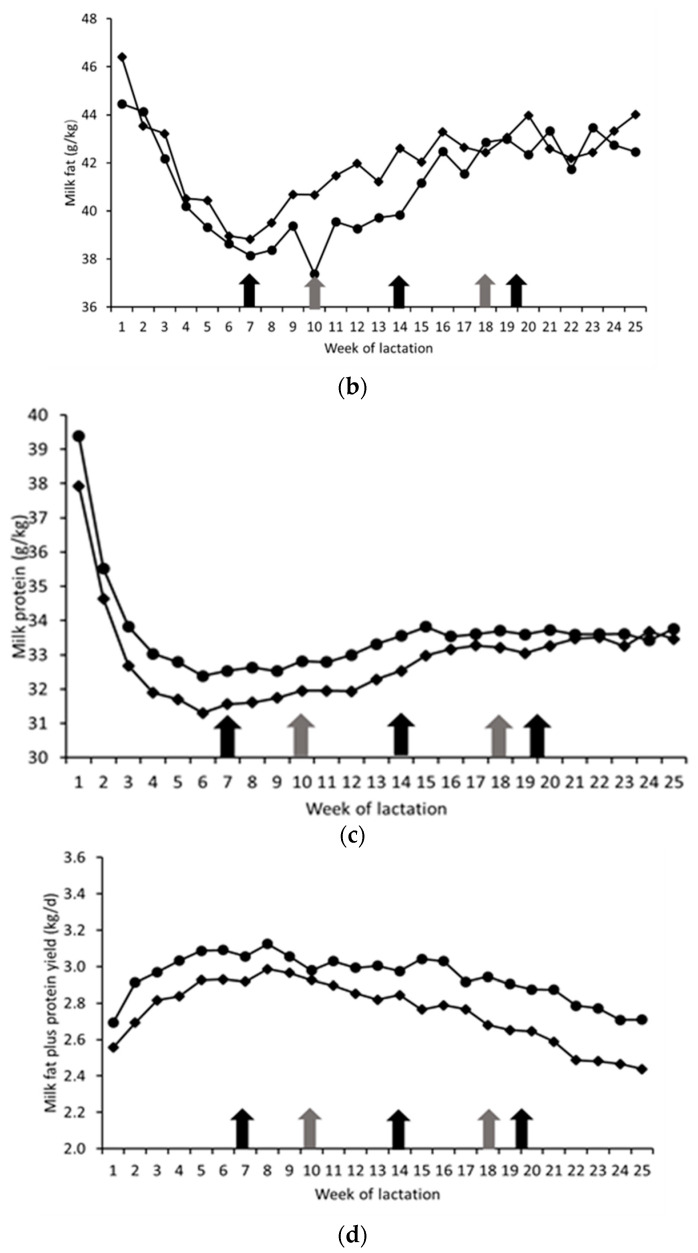
Mean weekly milk yield (kg/d) (**a**), milk protein composition (g/kg) (**c**), milk fat composition g/kg (**b**), and fat plus protein yield (kg/d) (**d**) of cows offered silages produced within either a three- (3H; ◆) or four-harvest (4H; •) system. Arrows indicate when cows changed to harvests 2,3 and 4 (4H; black arrow) and harvests 2 and 3 (3H; grey arrows).

Mean milk yield within each within harvest 1, 2 and 3 (3H treatment) were 40.3, 38.3 and 33.8 kg/day, while respective values within H1–H4 (4H system) were 40.7, 42.0, 39.2 and 36.1 kg/day. Respective values across harvests were 41.3, 42.4 and 43.0 g/kg (3H) and 40.8, 38.9, 42.2 and 42.5 (4H) for milk fat content, 32.6, 32.6 and 33.3 g/kg (3H) and 34.1, 33.0, 33.7 and 33.7 (4H) for milk protein content, 2.95, 2.84 and 2.56 g/kg (3H) and 3.04, 2.99, 2.94 and 2.72 (4H) for milk fat plus protein content. On average, cows on 4H treatment had a higher milk yield (*p* = 0.009), fat yield (*p* = 0.002), protein yield (*p* < 0.001) and fat + protein yield (*p* < 0.001) and produced milk with a higher protein content (*p* = 0.004) than those on 3H, while cows on 3H produced milk with a higher fat content (*p* = 0.002; Table 3). Milk yield, milk fat content, milk protien content and milk fat plus protein yield varied (*p* > 0.001) over the experimental period (Figure 2a–d, respectively). There were no interactions between treatment and week of lactation for any milk production parameter (*p* < 0.05).

Treatment had no effect on BW (average 637 kg) or BCS (average 2.5) over the experimental period, or on nadir BW or days to reach nadir BW (average 62 days). Cow BW and BCS varied over time (Table 4; *p* < 0.001), but there was no significant interaction between treatment and week of lactation. Mean calculated EB over the experimental period was 5.6 and 0.6 MJ/d for cows on 4H and 3H, respectively (*p* < 0.030; Table 4). While EB was affected by week of lactation (*p* < 0.001), there was no significant interaction between treatment and lactation week.

Treatment had no effect on plasma glucose levels (mean 3.49 mM/L). However, cows on 4H tended to have lower serum βHB and higher serum NEFA concentrations (*p* = 0.085 and *p* = 0.070, respectively; Table 4) compared to cows on 3H. Serum urea was significantly higher in cows on 4H compared to cows on 3H (*p* = 0.032). All blood metabolites were affected by week of lactation (*p* < 0.001) and there was an interaction between treatment and week for serum βHB and serum urea (*p* = 0.034, *p* < 0.001, respectively; Table 4).

## 4. Discussion

This study examined the performance of dairy cows offered silages produced within either a three or four harvest system, when supplemented with concentrates using an FTY approach. Feed-to-yield was adopted as this is normal practice on many NI dairy farms, and as responses to concentrate feeding are known to differ between FTY and flat-rate approaches assumptions cannot be made from previous work. The decision to offer concentrates specifically formulated to complement each silage type again reflects best practice, and the trajectory of modern rationing programmes. While individual ingredients differed between some of the concentrates offered, these were designed to supply the cows ME and MP requirements, which are the key drivers of cow performance, rather than ingredient type per se.

### 4.1. Impact on Silage Composition and Feed Value

Silage DM concentrations were variable (225–491 g/kg DM), with concentrations above target (300 g/kg DM at ensiling) arising due to excellent weather conditions and unexpected machinery related delays, while concentrations below target highlight the limitations of a 24 h period of field wilting within a temperate maritime climate, which is often associated with frequent rainfall events and high humidity. Silages were generally well fermented, with lactic acid concentrations and pH largely a function of DM content. Silage CP concentration tended to increase, and ME to decrease, with later harvests, agreeing with the trends identified within a 20 year dataset of NI silages [13]. Within this study the 4H silages had greater nutritive value compared to the 3H silages. While there will be year-to-year variation in yields and silage quality, the improved quality of the 4H silages reflect the earlier cutting and shorter re-growth intervals adopted. In general, ammonia N concentration, an indicator of the extent of deamination of plant protein by both plant and microbial enzymes, was lower in drier silages, and increased with increasing CP content in later harvests.

### 4.2. Impact on Cow Intakes and Performance

Comparisons between each harvest within the current study were not meaningful, due to the confounding effects of differences in lactation stages when each harvest was offered, and the absence of a fourth harvest within the 3H system. Thus, average performance over the entire study period was compared, with individual harvest data presented within Figure 1 and Figure 2 to aid the interpretation of the outcomes.

The weighted ME composition of the silages produced within the 4H system was 0.55 MJ/kg DM higher than for silages produced within the 3H system, equivalent to an increase in D-value of 34 g/kg. This increase would be expected to increase silage DMI by 0.92 kg/day [14], with this similar to the observed increase of 0.9 kg/d, thus demonstrating the benefits of more frequent harvesting. However, differences in intakes were inconsistent between treatments, with intakes of H1, H2 and H3 silages within the 4H system differing by +0.8 kg, −0.6 kg and +1.5 kg DM/cow/day, compared to the equivalent harvests within the 3H system (Figure 1). Silage composition data provides no obvious explanation for these inconsistencies.

Total DMI did not differ between treatments, with the higher silage intakes with 4H compensated for in part by a numerically lower concentrate DMI, reflecting the FTY feeding approach adopted in the study. The total DMI curves followed largely identical patterns until approximately week 18 of lactation, when intakes with the 3H treatment declined more rapidly than those of the 4H treatment, coinciding with the change to the third harvest of 3H which was the wettest silage within the study.

Difference in milk yield between treatments became apparent shortly after calving, milk yields on 3H remaining lower than for 4H throughout the study (mean difference over the experiment of 2.4 kg/day). This difference was larger than the response of 1.5 kg/day that might have been expected (0.45 kg milk per 10 g/kg increase in D-value [14]). Both milk fat and milk protein content exhibited the normal early lactation decline, with concentrations beginning to increase from approximately week six–seven of lactation onwards. The increased milk protein content with 4H (0.7 g/kg greater) was similar to the 0.5 g/kg increase expected based on Rinne et al. [15], namely a 0.14 g/kg increase in milk protein for each 10 g/kg increase in silage D-value. Similarly, in a review of 23 comparisons a mean increase in milk protein content of 0.09 g/kg for each 10 g/kg increase in D-value was observed [1]. The increase in milk protein content likely reflects an improved supply of MP to the small intestine. The lower milk fat content with 4H (1.0 g/kg lower) appeared to arise between week 7–19 of lactation, when silages from harvests two and three were offered. At this time, cows on both treatments were generally in positive EB, and consequently differences are likely to have been driven more by the lower NDF content of the 4H diets, rather than differences in tissue mobilisation. The cumulative effect of the differences in milk yield and milk composition was that milk fat plus protein yield was 0.2 kg/day higher in 4H cows compared to 3H cows. This improvement in milk production performance is likely due to the numerically higher total DMI with the 4H system, and the higher nutritive value of the 4H silages offered, which resulted in an 8 MJ/cow/day higher ME intake with this treatment. Differences in body tissue mobilisation between treatments appear to have been small, as evidenced by the absence of treatment effects on BW and BCS, and on βHB and NEFA concentrations, both of which can be indicators of body tissue mobilisation. Concentrations of both NEFA and βHB remained below 0.7 mM/L, and 1.0 mM/L, respectively, the maximum value suggested as normal [16]. Similarly, plasma glucose concentrations increased during the weeks post-calving, but did not differ between treatments, and remained above the optimal value of 3.0 mM/L [16].

These trends in body tissue reserves and blood metabolites are supported by the calculated EB of cows on the 4H and 3H treatments (5.6 vs. 0.6 MJ/day ME), which were numerically similar, although significantly different (*p* = 0.030). The magnitude of this difference is unlikely to be of biological importance, with cows on both treatments returning to positive EB at approximately week five of lactation. That energy balance of cows on both the 3H and 4H treatments were similar and this indicates that the FTY approach adopted was effective in meeting the calculated nutrient requirements of the cows at each performance levels.

Blood serum urea was significantly higher in 4H compared to 3H cows (4.44 vs. 4.15 mM/L), except at week 12 when 3H cows had elevated serum urea. The generally higher urea levels with 4H suggest an excess of effective rumen degradable protein relative to fermentable ME [16], with this likely to reflect the higher CP levels of the 4H silages (143 vs. 164 g/kg DM). Similarly, total diet CP levels, weighted across all diets offered, was higher in 4H compared to 3H diets, namely 179 and 171 g/kg DM, respectively. However, nitrogen utilisation efficiency (NUE) was similar with the 3H and 4H treatments, namely 0.32 and 0.33, respectively.

### 4.3. Whole Systems Comparison and Practical Considerations

Given the differences in herbage yield, DMI and cow performance between the two treatments, it is important to examine the impact of increasing the number of harvests from a whole systems perspective. The following analysis examined inputs and outputs associated with feeding a 100-cow dairy herd over a 180 day winter housed period. While the analysis is based on a single years herbage yield data, the 1.1 t DM/ha reduction in yield with the 4H compared to the 3H system (12.3 vs. 13.4 t DM/ha, respectively) is in agreement with the outcome of earlier AFBI studies indicating reduced yield with more frequent harvesting [5,17]. Assuming in-silo and feed-out losses of 15% within both systems, the utilisable silage yields available with the current 3H and 4H systems are 11.4 and 10.5 t DM/ha, respectively. Based on measured silage intakes within the current study, the total silage requirement over the 180 day housed period is 171 t and 187 t DM for the 3H and 4H systems, respectively (daily intake × herd size × housed period), a 9.5% higher requirement with the latter. Dividing the total silage requirement associated with each system (t DM) by the utilisable silage yield available within each system (t DM/ha), indicates a land requirement of 15 and 18 ha to supply the winter feed requirements of the 3H and 4H system respectively, a 19.3% higher requirement with the latter. The greater land requirement associated with the 4H system may be an issue in situations where land is a limiting resource. However, based on concentrate intake and milk output values in the current study, the 4H system requires 2.2% less concentrates (241 vs. 236 t DM for 3H and 4H system, respectively), and is associated with the production of a 6.9% higher yield of fat plus protein (49.5 vs. 52.9 t for 3H and 4H systems, respectively). The relative economic performance of these different approaches for a farmer will depend on a range of factors, including land value, silage production costs and concentrate costs, and the milk price received.

While a four-harvest silage system offers opportunities to reduce concentrate inputs and to increase milk solid output, the system is not without its challenges. To produce high digestibility silages, herbage must be harvested within a relatively short time-window, which can be challenging given the high reliance on contractor usage in many countries (>60% in NI [6]). Secondly, in order to achieve satisfactory fermentation, which can be challenging given the higher N content and possibly high nitrate levels in herbage, adequate wilting is necessary. However, unlike many parts of Europe, where the summer climate is influenced by continental air masses and tend to be relatively stable, western areas of the UK and the Ireland have a temperate maritime climate, with a significant proportion of annual rainfall between the months of April to September. Therefore, the short-time window for harvesting to ensure high digestibility must also be associated with weather conditions when wilting can be achieved. However, herbage harvested within a four-harvest system will have a faster rate of wilting than herbage harvested within a three-harvest system due to the lower herbage yield at each harvest.

## 5. Conclusions

Increasing the harvesting frequency of grass for silage production from three- to four-harvests per season has potential to improve silage feed value, and the performance (increased silage DMI, milk yield and milk fat plus protein yield) of cows offered concentrates using an FTY approach. While based on a single growth season, within the current study the lower DM yield (t/ha) with the four-harvest system would increase land area required by 19.3% compared to a three-harvest system. However, this was accompanied by a 2.2% reduction in concentrate use and a 5.9% increase in milk solids output. Therefore, producing grass silage within a four-harvest system is an effective way to improve silage feed value and increasing milk production per cow, although the economic benefits of adopting this approach will be dependent on a wide range of factors.

## Figures and Tables

**Table 1:** Ingredient list (g/100 g fresh) and chemical composition (SD in parenthesis) of the concentrate offered to all cows through the out-of-parlour feeding system (OPF), and of individual concentrates used to supplement each silage type within the three- (3H) and four-harvest (4H) silage production systems.

	Concentrate via OPF	3 Harvest System (3H)			4 Harvest System (4H)			
		Harvest 1	Harvest 2	Harvest 3	Harvest 1	Harvest 2	Harvest 3	Harvest 4
Ingredients								
Maize meal	12.6	25.8	44.1	44.6	30.7	45	44.6	44.6
Wheat		22.2	18.4	17		20	10	10.5
Barley		7.1	10	10				
Rapeseed meal	12.5				18.5	5	11.5	5
Soyabean meal (high protein)	6.4	16.6	5	5	14	5	5	5
Maize gluten	15	11.3	7.6	8.5				
Distillers dried grains with solubles	10	11	5	5				
Soya hulls (toasted)	15.75				26.6	15.1	19	25
Wheat feed meal	11							
Palm kernel meal	9.5							
Molaferm ^1^	4							
Protected protein (Sopralin) ^2^			3.75	3.75	4	3.75	3.75	3.75
Protected fat (Maxfat) ^2^		2.5	2.5	2.5	2.5	2.5	2.5	2.5
Limestone (CaCO_3_)	1.3	1.25	1.25	1.25	1.25	1.25	1.25	1.25
Rumen buffer (Acid buff) ^3^	0.6	0.89	1	1	1.05	1	1	1
Sodium chloride	0.65	0.65	0.65	0.65	0.65	0.65	0.65	0.65
Mineral and vitamin pre-mix ^2^	0.4	0.4	0.4	0.4	0.4	0.4	0.4	0.4
Calcined magnesite	0.3	0.3	0.3	0.3	0.3	0.3	0.3	0.3
Yeast (Actisaf) ^4^		0.04	0.05	0.05	0.06	0.05	0.05	0.05
Chemical Composition								
Oven dry matter (g/kg)	890 (16.1)	905 (4.1)	898 (6.4)	897 (6.2)	906 (9.5)	892 (11.8)	898 (5.2)	899 (5.8)
Starch (g/kg DM)	151 (27.8)	384 (28.0)	437 (31.6)	457 (18.2)	220 (20.1)	401 (23.3)	363 (8.8)	359 (32.7)
Crude protein (g/kg DM)	211 (5.7)	205 (4.0)	156 (8.2)	164 (4.8)	230 (7.8)	163 (15.6)	169 (5.2)	150 (6.6)
ADF (g/kg DM)	199 (16.5)	61 (3.8)	56 (12.4)	56 (5.0)	186 (16.4)	125 (14.3)	143 (13.9)	169 (23.9)
NDF (g/kg DM)	421 (30.5)	219 (5.8)	191 (16.2)	196 (17.0)	361 (17.3)	260 (17.9)	281 (29.3)	321 (38.0)
Ash (g/kg DM)	88 (2.4)	75 (4.3)	65 (2.5)	67 (2.7)	83 (5.2)	77 (8.1)	75 (2.1)	73 (4.3)
Metabolisable energy (MJ/kg DM)	12.0	13.3	13.5	13.5	12.6	13.2	12.9	13.0

^1^ United molasses, Belfast, UK. ^2^ Trouw Nutrition, Belfast, UK. ^3^ Celtic Sea Minerals, Cork, Ireland. ^4^ Lesaffre, Marcq-en-Baroeul, France.

**Table 2:** Dry matter of herbage at ensiling, and the chemical composition (SD in parenthesis) of the resultant silage as produced within a three- (3H) or four-harvest (4H) system.

	3 Harvest System (3H)			4 Harvest System (4H)			
	Harvest 1	Harvest 2	Harvest 3	Harvest 1	Harvest 2	Harvest 3	Harvest 4
Herbage pre-ensiling							
Oven dry matter (g/kg)	403 (37.4)	289 (36.1)	234 (29.4)	322 (53.1)	513 (62.2)	263 (45.4)	266 (15.7)
Silage							
Oven dry matter (g/kg)	387 (29.4)	282 (29.6)	225 (20.6)	279 (16.8)	491 (33.1)	261 (31.8)	252 (14.9)
VCODM (g/kg)	400 (40.5)	292 (18.2)	239 (18.7)	296 (12.7)	505 (14.1)	275(12.7)	269 (10.5)
Crude protein (g/kg DM)	106 (9.4)	161 (19)	176 (19)	124 (9.8)	164 (14.2)	180 (9.5)	203 (8.7)
Ash (g/kg DM)	70 (1.9)	92 (5.5)	125 (5.1)	74 (1.7)	90 (4.0)	110 (3.0)	125 (6.1)
Acid detergent fibre (g/kg DM)	326 (7.5)	289 (14.9)	286 (10.0)	264 (4.6)	287 (6.5)	265 (11.2)	249 (14.1)
Neutral detergent fibre (g/kg DM)	562 (7.9)	504 (28.1)	476 (15.2)	469 (13.2)	505 (18.6)	474 (28.5)	432 (21.0)
Gross energy (MJ/kg DM)	18.7 (1.18)	19.1 (0.64)	18.5 (0.99)	19.1 (1.17)	19.2 (0.38)	18.4 (0.67)	18.5 (0.55)
Metabolisable energy (MJ/kg DM)	10.9 (0.36)	10.6 (0.52)	10.6 (0.38)	12.1 (0.39)	11.2 (0.50)	10.7 (0.39)	10.8 (0.47)
pH	4.34 (0.19)	4.07 (0.16)	4.14 (0.23)	3.73 (0.21)	4.85 (0.16)	4.04 (0.11)	4.21 (0.22)
Lactic acid (g/kg DM)	46 (12.1)	66 (34.2)	89 (53.4)	127 (32.0)	20 (7.5)	101 (26.9)	106 (42.4)
Acetic acid (g/kg DM)	10.3 (3.34)	28.7 (11.25)	39.2 (18.62)	15.4 (3.41)	7.3 (1.74)	13.3 (3.07)	22.0 (4.14)
Ethanol (g/kg DM)	5.7 (3.04)	5.5 (1.68)	6.2 (3.27)	18.1 (10.12)	5.7 (2.75)	10.1 (4.60)	7.3 (3.83)
Ammonia (g/kg total N)	66 (1.2)	71 (0.5)	86 (1.9)	58 (1.2)	49 (0.6)	66 (0.5)	80 (2.2)

VCODM, volatile corrected oven dry matter.

**Table 3:** Effect of offering silage made within either a three- (3H) or four-harvest (4H) system on average daily intakes, milk production and milk composition across all harvests.

	Treatment			*p* Value		
	3 Harvest System (3H)	4 Harvest System (4H)	SED	Treatment (d.f. 1)	Week (d.f. 24)	Treatment × Week (d.f. 24)
Silage DMI (kg/day)	9.5	10.4	0.30	<0.001	<0.001	<0.001
Concentrate DMI (kg/day)	13.4	13.1	0.43	0.165	<0.001	0.123
Total DMI (kg/day)	23.0	23.4	0.59	0.131	<0.001	0.172
Milk yield (kg/day)	37.3	39.7	1.08	0.009	<0.001	0.501
Fat (g/kg)	42.1	41.1	1.28	0.022	<0.001	0.587
Protein (g/kg)	32.9	33.6	0.45	0.004	<0.001	0.140
Lactose (g/kg)	48.3	48.2	0.32	0.178	<0.001	0.737
Fat yield (kg/day)	1.54	1.61	0.058	0.002	<0.001	0.416
Protein yield (kg/day)	1.22	1.32	0.038	<0.001	<0.001	0.524
Fat plus protein yield (kg/day)	2.75	2.94	0.087	<0.001	<0.001	0.516

DMI: dry matter intake.

**Table 4:** Effect of offering silage made within either a three- (3H) or four-harvest (4H) system on cow body weight, body condition score and mean energy balance over the experimental period, and on mean blood metabolites.

	Treatment			*p* Values		
	3 Harvest System (3H)	4 Harvest System (4H)	SED	Treatment (d.f. 1)	Week (d.f. 24)	Treatment × Week (d.f. 24)
Bodyweight (kg) ^1^	637	636	9.6	0.788	<0.001	0.349
Nadir bodyweight (kg)	613	609	11.5	0.721		
Days to nadir body weight	63	61	12.1	0.871		
Body condition score ^1^	2.5	2.4	0.05	0.792	<0.001	0.362
End of study body condition score	2.5	2.5	0.08	0.916		
Energy balance (MJ/day) ^1^	5.6	0.6	0.23	0.030	<0.001	0.148
Blood metabolites ^2^						
βHB (mM/L)	0.43	0.39	0.034	0.085	<0.001	0.034
NEFA (mM/L)	0.16	0.18	0.020	0.070	<0.001	0.150
Glucose (mM/L)	3.52	3.46	0.327	0.679	<0.001	0.550
Urea (mM/L)	4.15	4.44	0.184	0.032	<0.001	<0.001

^1^ Mean across entire experimental period. ^2^ Mean analysis of samples taken at 4, 8, 12, 16 and 20 weeks post-calving. βHB, beta-hydroxybutyrate; NEFA, non-esterified fatty acid.

## Data Availability

None of the data were deposited in an official repository.
